# General practitioners’ and nurses’ views on medication reviews and potentially inappropriate medicines in elderly patients – a qualitative study of reports by educating pharmacists

**DOI:** 10.1080/02813432.2018.1487458

**Published:** 2018-06-29

**Authors:** K. Schmidt-Mende, J. Hasselström, B. Wettermark, M. Andersen, P. Bastholm-Rahmner

**Affiliations:** a Academic primary health care centre, Stockholm County Council and Division of Family Medicine and Primary Care, Department of Neurobiology, Care Sciences and Society, Karolinska Institute, Huddinge, Sweden;; b Public Healthcare Services Committee, Stockholm County Council, Stockholm, Sweden;; c Centre for Pharmacoepidemiology, Department of Medicine Solna, Karolinska Institute, Karolinska University Hospital, Stockholm, Sweden;; d Department of Drug Design and Pharmacology Faculty of Health and Medical Sciences, University of Copenhagen, Copenhagen, Denmark;; e Research Unit of General Practice, Department of Public Health, University of Southern Denmark, Odense, Denmark;; f Medical Management Center (MMC), Department of Learning, Informatics, Management and Ethics (LIME), Karolinska Institute, Stockholm, Sweden

**Keywords:** Inappropriate prescribing, medication reviews, aged, primary health care, qualitative study

## Abstract

**Objective:** The aim with this study was to understand more about how general practitioners (GPs) and nurses in primary care experience their work with medication reviews in elderly patients.

**Design:** This qualitative study was nested within a cluster randomised trial and analysed narrative and unstructured diaries written by two pharmacists who performed academic detailing, i.e. educational outreach visits in primary care. The educational sessions dealt with potentially inappropriate medicines, and stimulated interprofessional dialogue in relation to medication reviews. The purpose of the diaries was to document and structure the pedagogical process of academic detailing and contained quotes from 194 GP and 113 nurse participants in the sessions, and the pharmacists’ reflections. The data was explored using thematic analysis.

**Setting:** Thirty-three primary care practices in Stockholm, Sweden.

**Subjects:** GPs and nurses working in primary care.

**Main outcome measures:** Thematic descriptions of academic detailing by pharmacists.

**Results:** Five themes were identified: 1) Complexity in 3 ‘P’: patients, pharmacotherapy, and primary care; 2) What, when, who? Clash between GPs’ and nurses’ experiences and guidelines; 3) Real-world problems and less-than-ideal solutions; 4) Eureka? Experiences with different steps during a medication review; and 5) Threats to GP autonomy.

**Conclusion:** GPs and nurses should participate in the construction and release of guidelines in order to increase their usability in clinical practice. Future research should analyse if alternative strategies such as condensed medical reviews and feedback on prescribing are easier to implement in primary care.

**Key points**
- Complex medication reviews have been introduced on a large scale in Swedish primary care, but knowledge on GPs’ and nurses’ views on such reviews is lacking.
- In the context of primary care alternative strategies such as condensed medication reviews and feedback on prescribing may be more applicable than medication reviews according to guidelines.
- GPs and nurses should make contributions to the development of guidelines on medication reviews in order to increase their usability in clinical practice.

## Introduction

Elderly people often suffer from several diseases and are treated with many drugs. As the number of drugs increases, so does the subsequent risk for adverse drug reactions [1]. Adverse drug effects may cause up to 15% of unplanned hospitalizations in the elderly population [2]. One possible measure to improve drug treatment and make it more appropriate for the individual patient is medication reviews (MRs) [3]. In many cases MRs are performed by a team of pharmacist and physician. During a MR, the appropriateness of drug treatment is assessed in relation to professional experience or validated criteria of potentially inappropriate medicines (PIMs) such as Irish STOPP/START criteria [4], Swedish ‘National indicators for quality of drug therapy in elderly persons’ or Norwegian NORGEP criteria [5]. Those criteria include for example drug-drug interactions and ‘drugs to avoid’ such as long acting benzodiazepines. However, the manner in which MRs are performed differs between studies or is not sufficiently described. This may partly be due to the fact that it is unclear which aspects of a MR are most important [6]. Moreover, the effects of MRs are unclear [3]. Hospital-based MRs performed in collaboration between physician and pharmacist may reduce drug-related hospital admissions [7]. Effects of MRs performed in primary care have only rarely been analysed systematically. Two studies performed in Swedish primary care did not show a reduction of drug-related problems or PIM use after MRs [8] [9].

GPs have a central and connecting role in the health care system. In Sweden, elderly people see their GP on average four times a year. Still, Swedish primary care does not have a gatekeeper function, and patients may seek specialist care without referral. Patients may therefore fill prescriptions from many different health care providers. At the same time, there is no consensus among GPs regarding their level of responsibility for the patients’ drug list [10], which complicates the management of medical care in elderly people. Other barriers to minimize the use of PIMs are GPs feeling pressure from patients to prescribe [11], lack of time and resources [12], and a suboptimal communication between primary and secondary care [13]. GPs expressed helplessness in relation to polypharmacy in their elderly patients and demand for simple measures to reduce the use of PIMs [14].

In 2012, the Swedish National Board of Health and Welfare revised the guidelines on ‘basic’ and ‘complex’ MRs in patients 75 years and older with at least five drugs performed in primary and secondary care (supplementary file) [15]. A financial incentive of 300 SEK ($35) was given by Stockholm County Council for every basic MR performed in primary care and registered in the electronic patient record. Moreover, there was a penalty for the practice if not at least 50% of home care patients had been registered with a basic MR. The aim with this study was to understand more about general practitioners’ (GPs) and nurses’ views on PIMs and MRs in elderly patients.

## Material and methods

### Study design and data collection

This was a qualitative study analysing narrative and unstructured diaries written by two pharmacists (hereafter called tutors) who performed educational outreach visits in primary care. Diary writing is an important data collection method in qualitative research and may stimulate the creation of hypotheses and help to identify problems [16]. A qualitative approach was chosen because very little was known about GPs’ and nurses’ experiences with MRs.

The educational outreach visits were part of a previous cluster randomised controlled trial [17]. We designed this multifaceted educational intervention based on the ‘National indicators for quality of drug therapy in elderly persons’ [18] and the new guidelines on MRs (supplementary file) [15]. The trial did neither reduced the use of PIMs nor subsequent acute health care consumption in elderly patients [17]. One aim of the randomised trial had been to assist GPs and nurses in developing a working procedure for complex MRs according to the guidelines (supplementary file) [15]. The tutors worked for the regional drug and therapeutics committee and had several years of experience with academic detailing in primary care [19]. The writing of diaries was a standardised part of their working procedures to document and structure the pedagogical process of academic detailing. Of note, the tutors were not steered in their reporting and thus had the maximum freedom of documenting whatever they considered to be important regarding their purpose (to document the pedagogical process). In the context of our cluster randomised trial [17], they had decided to do the same in all educational sessions. When the tutors presented their diaries for the research group, we discovered they contained unexpectedly rich and extensive data. As we were not aware of any study dealing with GPs’ and nurses’ views on MRs according to guidelines, we decided to analyse the diaries by a qualitative approach. The tutors consented to the analysis of the diaries. As GPs and nurses attending the educational sessions were not aware the diaries would be analysed we applied and obtained ethical approval to analyse them in addition to the earlier approval for the cluster randomised trial [17]. Moreover, GPs and nurses were anonymous to the researchers, and it was not possible to identify them based on the tutors’ diaries.

The educational sessions (1 - 2 hours) were directed to both nurses and GPs and were given in Swedish. Nurses have an important role in Swedish primary care and in relation to patients’ drug treatment. District nurses prescribe drugs to a minor extent (less than 1% of total prescriptions). Nurses regularly perform home visits in elderly patients. However, they work under the supervision of a GP [20]. District nurses often communicate patients questions and complaints raised by the patients to the GP and are suggested to actively monitor side effect of drugs. The contents of the educational sessions had been developed by two GPs, three pharmacists and a nurse. During the sessions, it was referred to the Swedish ‘National indicators for quality of drug therapy in elderly persons’ [18], and the legislated two types of MRs: basic and complex (supplementary file) [15]. The following steps were part of the sessions: First, the tutors gave a powerpoint-presentation on PIMs and MRs according to the guidelines. Practice specific feedback was provided based on prescription data extracted from the Central Regional Data Warehouse of Stockholm County [21], which contains data on health care consumption, drugs, migration, and deaths for all 2.1 million inhabitants of Stockholm County. For example, we provided feedback on how many patients aged 65 and older registered with the practice had received long-acting benzodiazepines. Second, the tutors asked GPs and nurses to create a working procedure on MRs by interprofessional dialogue. The tutors were present during this dialogue and could support the process. GPs and nurses shared each other’s responses and thus gave additional comments, developed and completed their answers. The working procedure was documented on an A1-poster which was set up in the practice as a reminder. Third, a second educational session four months later was provided, with repeated information on PIMs, written reminders of the first session as well as relevant internet links in relation to PIMs. Furthermore, GPs and nurses discussed with the tutors if and how the working procedure on MRs had been put into practice. Thus, the educational sessions gave the tutors access to how GPs and nurses were thinking about MRs and PIMs among elderly patients in the context of primary care.

The tutors wrote in total 40 pages of diaries directly after each educational session relying on their memories. No equipment was used to record the sessions. The tutors visited 33 primary care practices on their own twice within four months (Table 1). In total, 32/33 (97%) of the diaries on the first session, and 30/33 (91%) diaries on the second session were available, and the diaries contained quotes from 194 GPs and 113 nurses participating in the educational sessions (Table 1). The diaries contained three types of data. First, the tutors directly quoted the specific GPs and nurses who had participated in the sessions, for example: ‘Who is responsible for what? I can’t stop a medication initiated by another physician.’ Second, the tutors made their own inferences regarding specific GP or nurse comments, for example: “The physician responsible for home care patients implied that a 90 year old patient does not benefit from treatment with simvastatin.“ Third, the diaries reflected the tutors’ personal perceptions and experiences, for example: ‘though unspoken, there was a feeling that ‘we (the GPs) are doing this all the time… we have always done this’, at least in some people.’ Sometimes it was not clear from the diaries if the healthcare professional cited by the tutors was a GP or nurse.

**Table 1.** Basic characteristics of the 33 primary care practices receiving the educational intervention and where the tutors wrote diaries.

Variable	33 practices
Patients registered at practice	
Median number of registered patients aged ≥65/practice (min-max)	1 529 (634-3394)
Median % of patients aged ≥65/all registered patients (min-max)	15.1 (7.4-24.2)
Employment	
Median number of general practitioners/practice (min-max)	8.7 (2.2-18.3)
Median number of registered nurses/practice (min-max)	7.0 (2.0-12.0)
Participation in educational sessions	
General practitioners: total number; median (min-max)	
First session	194; 6 (min 1; max 17)
Second session	166; 5 (min 0; max 14)
Registered nurses: total number; median (min-max)	
First session	113; 4 (min 0; max 7)
Second session	92; 3 (min 0; max 9)

### Data analysis

A data driven inductive thematic analysis with no pre-determined categories was performed in a stepwise manner [19]. Thematic analysis permits to identify, explore and report patterns within data in rich detail [22], which is why we considered it to fit our aim but even the character of our data material. As there is a risk in qualitative research that preconceived notions influence the way researchers interpret the data, the analysing researchers KSM (GP) and PBR (behavioral scientist) documented their preconceived notions regarding PIMs and MRs prior to data assessment. This is a way to reduce” reflexivity “(=”sensitivity to the ways in which the researcher and the research process have shaped the collected data“, quotation from [23]) and increase the quality of data analysis. Data was anonymous with respect to practice, tutor and participants. During the first step of the analysis, we decided which topic each sentence or paragraph was dealing with. This topic was called a ‘code’, leading to a total of 21 codes. Examples for such codes are: ‘frustration’, or ‘responsibility for MR’. Next, diary quotes in each coded group were evaluated looking for a possible connection to diary quotes in another coded group. This step of analysis led to the identification of five independent themes. During the third and fourth step of the analysis, KSM and PBR sifted through the diary quotes, codes, themes and their potential relationship, until consensus was reached. An iterative process was used throughout the whole analysis, i.e. moving from the diaries to the condensed description and back again. Finally, a pattern of relationships between the five themes was established.

When there was consensus among GPs and nurses within a particular theme or subtheme, diary quotes are presented in plain text boxes (example: box 1). When GPs’ and nurses’ experiences differed from guidelines (example: box 2) or when GPs’ and nurses’ experiences varied (example: box 3), we present diary quotes in ‘variation thermometers’ illustrating the extremes of experiences at the top and bottom of the thermometer to the left side.

Box 1 Diary quotes being part of theme ‘Complexity in 3 ‘P’: patients, pharmacotherapy, and primary care’.

***1. Patients***

 • *These patients have several challenges beyond the complicated medical regimen*

 • *With reference to screening for side effects with a standardized questionnaire, one GP suggested that ‘all’ elderly patients experience vertigo.*

***2. Pharmacotherapy***

 • *The GP responsible for home care patients implied that a 90 year old patient does not benefit from treatment with simvastatin. Another GP meant that withdrawing a drug* treatment based on a patient’s age was discriminatory against elderly patients.

***3. The role of primary care in the health care system***

 • *Once I (tutor) had finished, a GP mentioned that medication reviews are time-consuming, and that those providing different educational sessions regard their particular topic as most relevant. In addition, expectations with regard to primary care continue to increase.*

 • *Too difficult to get in contact with physicians working in the hospital.*

**Box 2.**
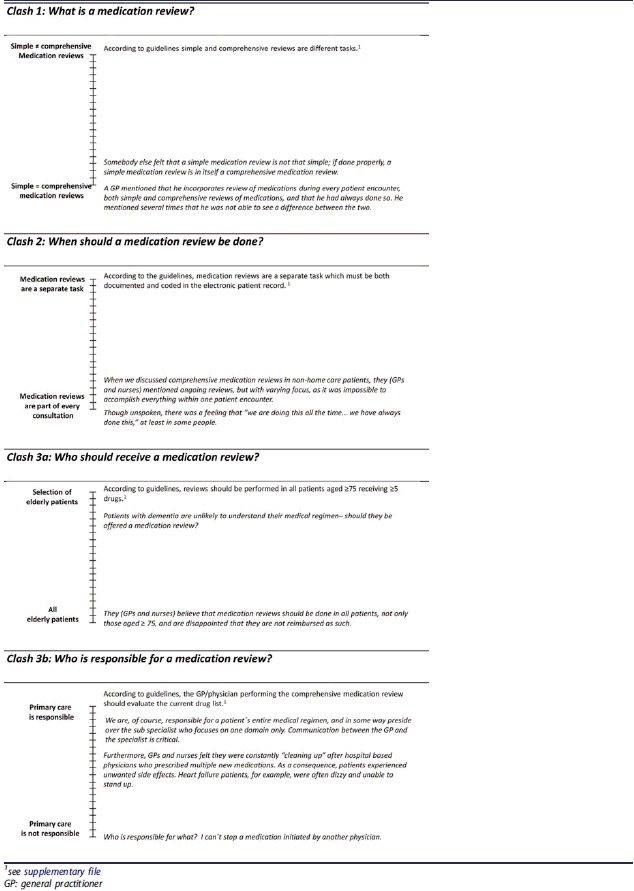
Diary quotes (italic style) and quotes derived from the guidelines on medication reviews (normal style) being part of theme ‘What, when, who? Clash between GPs´ and nurses´ experiences and the guidelines’. (Remark: When GPs´ and nurses´ experiences varied, diary quotes are presented as ‘variation thermometers’ illustrating the extremes of experiences at the top and bottom of the thermometer to the left side).

**Box 3.**
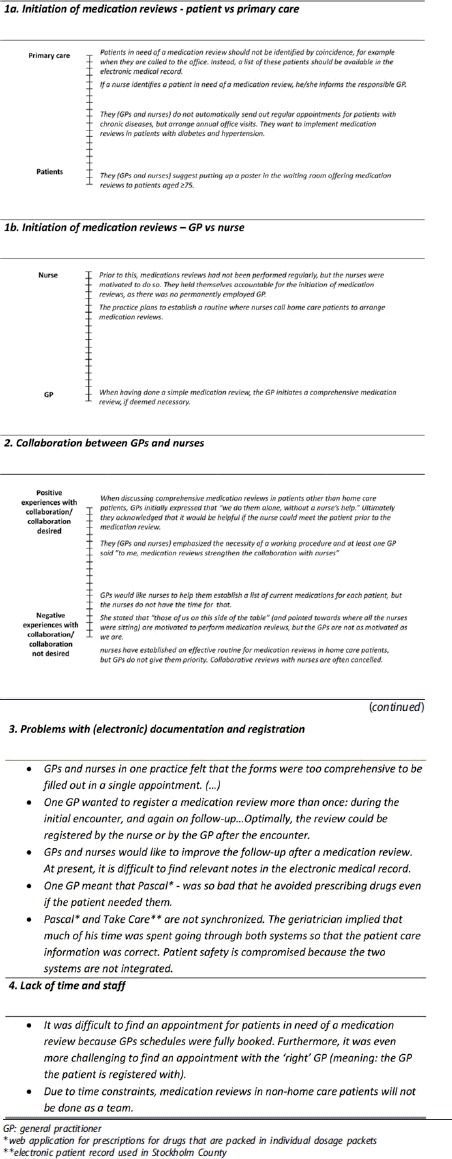
Diary quotes being part of theme ‘Real-world problems and less-than-ideal solutions’. (Remark: When GPs´ and nurses´ experiences varied, diary quotes are presented as ‘variation thermometers’ illustrating the extremes of experiences at the top and bottom of the thermometer to the left side).

### Ethical consideration

We obtained ethical approval for the qualitative analysis of the diaries (ethical board in Stockholm, DNR 2015/1927-32) in addition to earlier approval for the cluster randomised trial [16] (ethical board in Stockholm, DNR 2012/1266-31). The tutors’ diaries served as the main source for the analysis. We reported our research following the COREQ guidelines which is a checklist containing 32 criteria for reporting qualitative research [24].

## Results

GPs’ and nurses’ views on MRs as proposed by the guidelines and reported by the tutors could be grouped into five themes. 1) Complexity in 3 ‘P’: patients, pharmacotherapy, and primary care; 2) What, when, who? Clash between GPs’ and nurses’ experiences and guidelines; 3) Real-world problems and less-than-ideal solutions; 4) Eureka? Experiences with different steps during a medication review; and 5) Threats to GP autonomy.

### Theme 1: Complexity in 3 ‘P’: patients, pharmacotherapy, and primary care

There was consensus among GPs and nurses regarding the complexity of PIMs and MRs in elderly patients. Complexity referred to three topics: patients, pharmacotherapy and primary care (box 1).

First, elderly patients often had complex problems besides drug treatment. It was challenging to evaluate their symptoms, as chronic disease and side effects may cause the same symptoms making it difficult to differentiate between them. Second, pharmacotherapy was a complex subject. GPs often had to rely on their clinical experience when deprescribing drugs in elderly patients. There was consensus among GPs regarding the complexity of elderly patients’ drug treatment but they had different experiences on how to manage this complexity. Third, primary care had a complex role in the health care system and was responsible for multiple tasks, MRs in elderly patients being only one of them. Moreover, communication between primary care and healthcare professionals at hospitals was suboptimal.

### Theme 2: What, when, who? Clash between GPs’ and nurses’ experiences and the guidelines

GPs and nurses experiences with MRs differed in several ways from the guidelines. Three subthemes (=clashs), ‘what, when, who’ were identified (box 2).

First, in relation to ‘what is a MR’, there was a view of consensus that the separation into ‘basic’ and ‘complex’ MRs was arbitrary and did not correspond to the clinical experience of GPs and nurses actually involved in the care of elderly patients. Second, in relation to ‘when should a MR be done’, they perceived that MRs are a natural part of an everyday consultation, and that MRs therefore were done continuously and when needed. Third, the ‘who should receive a MR’ referred to patients in need of MRs. GPs and nurses doubted if patients in need of MRs should be identified upon their age. Fourth, the ‘who is responsible for a MR’ described that it is unclear if primary care has the overall responsibility for the patient’s current drug list, and to which extent a GP may change prescriptions issued by other specialists.

### Theme 3: Real-world problems and less-than-ideal solutions

In clinical practice, GPs and nurses may identify numerous ‘real-world’ problems when trying to implement new guidelines. Sometimes they are forced to find ‘less-than-ideal’ solutions because of unclear guidelines or organizational obstacles. The real-world problems and less-than-ideal solutions could be grouped in four subthemes (box 3).

First, there was variation in experiences regarding the practical solutions in relation to the initiation of MRs, ‘patient vs. primary care’ and ‘GP vs. nurse’. Some suggested that the practice should actively approach patients in need of a MR, while others thought that the patient should become active when interested in a MR. Sometimes nurses initiated MRs, whereas in other practices GPs initiated them. Second, there was variation regarding the GPs’ and nurses’ motivation to collaborate as well as their experiences regarding their collaboration. Some considered the collaboration as fruitful, whilst others experienced that the other part (GP or nurse) did not want to collaborate. Third, there was consensus regarding difficulties with the documentation and the registration of MRs. The forms to fill in during a MR were too complex, and it was unclear how to code correctly for MRs in order to be reimbursed. Furthermore, GPs and nurses agreed upon obstacles related to the electronic medical record. Important record notes were difficult to find, and synchronization between different electronic systems was suboptimal, both compromising patient safety. Fourth, shortage of health care professionals was a hinder for the conduct of MRs, as it was difficult to give the patient an appointment with his registered GP and as it was impossible to perform MRs in team of GP and nurse as claimed by the guidelines.

### Theme 4: Eureka? Experiences with different steps during a MR

GPs’ and nurses’ experiences with different steps during a MR as proposed by the guidelines were compiled in theme ‘Eureka? Experiences with MRs’ (box 4).

First, GPs’ and nurses’ experiences varied in relation to the usefulness of standardized questionnaires screening for side effects. Some experienced such questionnaires to be useful tools, whereas others thought that they caused more work and are difficult to interpret. Some even meant that patients would inform the GPs and nurses if they experienced side effects. Second, there was consensus among GPs and nurses regarding the value of calculating renal function. Third, GPs’ and nurses’ experiences regarding the usefulness of MRs in general varied. Some experienced that they were very useful, because they gave a more holistic picture of the patient’s medical regimen, and as many errors in the drug list were corrected. GPs and nurses at one practice were not able to identify any positive experiences with MRs and indicated that the concept itself was completely unclear to them.

**Box 4.**
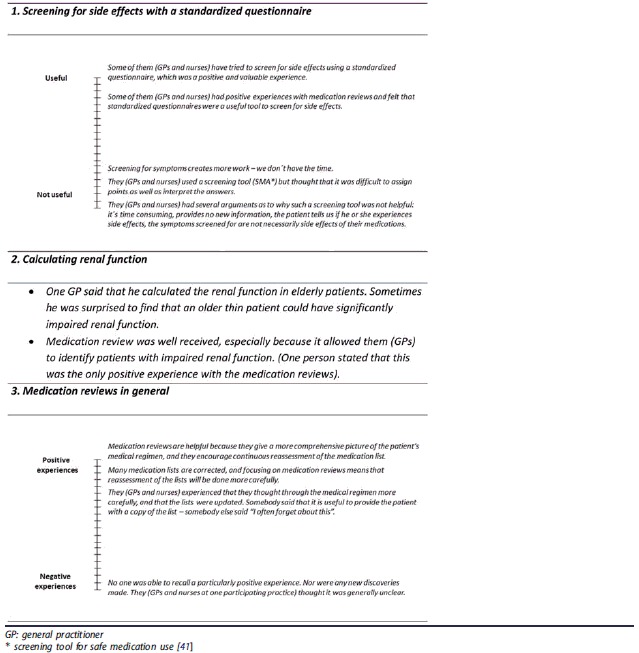
Diary quotes being part of theme ‘Eureka? Experiences with different steps during a medication review’. (Remark: When GPs´ and nurses´ experiences varied, diary quotes are presented as ‘variation thermometers’ illustrating the extremes of experiences at the top and bottom of the thermometer to the left side).

### Theme 5: Threats to GPs’ autonomy

This theme illustrated how external steering and financial incentives counteract GPs’ autonomy and their intention to offer the best medical care to their patients (box 5). There was consensus among GPs regarding both subthemes.

Box 5 Diary quotes being part of theme ‘Threats to GPs’ autonomy’.

***1. External steering***

 • *Now I (GP) will stop using my intuition and only follow checklists.*

 • *They (GPs and nurses) do not feel capable enough to establish a routine for medication review because the guidelines they must follow are too strenuous. At the same time, they do not want to cheat.*

 • *Some of us are provoked by your presentation since we currently do and have done those things in the past, but others understand that there are things we can learn and improve upon.*

***2. Financial incentives***

 • *In home care patients it is not always meaningful to do reviews (for example the patients are recently discharged from the hospital where a medication review was done), but it must be repeated and coded again, otherwise we are penalized. In those situations reviews, are a waste of time.*

 • *The question remains—how can we possibly focus on the patients most in need of a review, if the less acute patients must often be prioritized in order to be reimbursed properly?*

GP: general practitioner

First, external steering as represented by the guidelines (supplementary file) caused frustration and even passiveness, as they challenged GPs’ capacity to correctly evaluate a patient’s medical complaints. Second, GPs and nurses perceived that financial incentives forced them to perform MRs when they were actually not indicated from a medical point of view, which even made them lose time for those patients who were in need of their help.

### The relationship between the five themes

**Figure 1.**
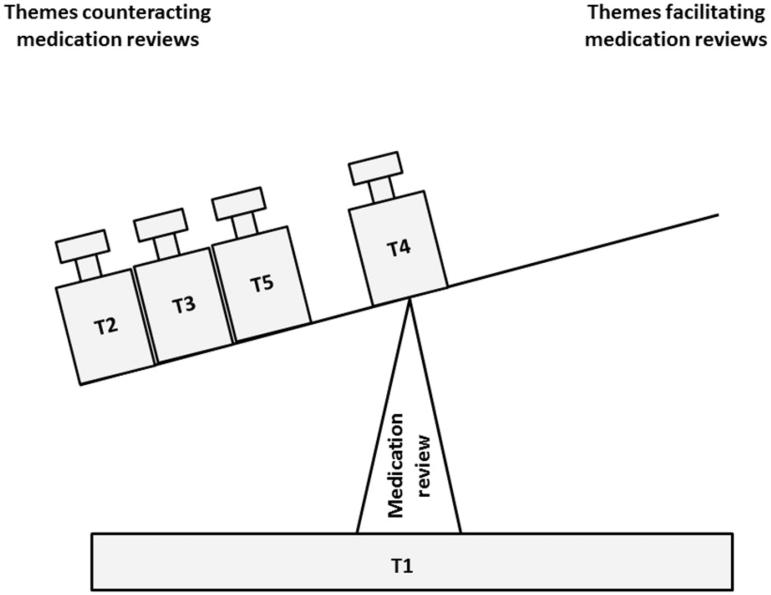
Visualisation of the imbalance between factors (= balance weights = themes = T) counteracting and facilitating the performance of medication reviews. The figure was created based on GPs´ and nurses´ expressed views. Of note, T2, T3 and T5 have been placed on the balance in a random order as we consider them to have the same weight. Abbreviations: T1: Complexity in 3 ´P´: patients, pharmacotherapy, and primary care; T2: What, when, who? Clash between GPs´ and nurses´ experiences and the guidelines; T3: Real-world problems and less-than-ideal solutions; T4: Eureka? Experiences with different steps during a medication review; T5: Threats to GPs´ autonomy.

The five themes related to each other as illustrated in Figure 1. The complexity of PIMs and MRs as proposed by the guidelines is represented by the bottom of the balance. MRs are a possible measure to reduce PIMs. However, GPs and nurses expressed that the factors (=balance weights = themes) complicating the performance of such MRs outweighed those facilitating their performance (Figure 1).

## Discussion

GPs’ and nurses’ experiences with MRs could be grouped into five themes and 15 subthemes. There was consensus among GPs and nurses regarding the complexity of the clinical evaluation of elderly patients and their drug treatment (theme 1) as well as the effect of external steering and financial incentives on GPs’ autonomy (theme 5). GPs and nurses disagreed with several aspects of the guidelines’ definition of MRs (theme 2). Due to organizational obstacles they were at times forced to choose suboptimal solutions when implementing the guideline on MRs (theme 3). GPs’ and nurses’ experiences regarding different steps during a MR varied (theme 4). Factors complicating the performance of MRs as proposed by the guidelines outweighed those facilitating their performance.

In many cases diaries are not created by the subjects who are the focus of the research [16], as was the case even in our study where pharmacists documented GPs’ and nurses’ experiences. Still, diaries may be an important and valuable data source in qualitative research as they help researchers to learn more about the participants’ views [16]. A weakness with diaries is that they, contrary to semi-structured interviews, do not allow to gain a deeper understanding. Another weakness is that the dialogues during the educational sessions were not recorded, which makes it difficult to evaluate if the tutors reported selectively. Still, this seems unlikely as the diaries were written with the purpose to memorize what had been said and expressed by the participants thus documenting the pedagogical process and allowing a natural starting point for the follow up session; reporting selectively would thus have impeded the usefulness of the diaries for the tutors.

We performed a secondary analysis of qualitative data, meaning that the data material served to answer a research question that differs from the original purpose of data collection [25]. Secondary analysis of qualitative data has been described as a valuable research method, if the researcher is aware of the strengths and limitations of such analyses [25]. Therefore, the results were validated by three GPs and two nurses who had not participated in the educational sessions. They recognized themselves in the themes and subthemes. However, one GP experienced that there were more positive experiences with MRs among GPs and nurses in clinical practice than we have captured in this study. This would imply that the balance in Figure 1 may tilt over more to the right than our data suggest, i.e. themes facilitating MRs are more balanced. A possible explanation for this finding may be that the tutors were pharmacists and spokesmen for MRs as proposed by the guidelines (supplementary file), which may have enhanced GPs’ and nurses’ resistance to the message the tutors delivered. Another explanation may be that GPs and nurses partly commented on MRs as part of an implemented control system even including financial incentives rather than MRs as a medical procedure. Still, it is a long tradition in Stockholm County that pharmacists from the regional drug and therapeutics committee perform educational outreach visits in primary care [19], and healthcare professionals respect the role of educating pharmacists. This committee is well recognised by GPs and nurses as a source of independent drug information, and educational outreach visits performed by the committee are an established way of implementing knowledge in primary care. The Drug committee may thus be considered as one of the ***”***addressers***“ ***of the medical procedure on MRs. We therefore think that the negative experiences GPs and nurses described in our study rather refer to the medical procedure than the implemented control system.

MRs as proposed by the guidelines (supplementary file) are complicated to perform as they involve both the collaboration between the patient and different healthcare professionals as well as the screening for multiple inappropriate drugs or drug combinations. GPs and nurses experience that such procedure is difficult to implement in clinical practice (box 5). This result is consistent with previous studies where clinicians have expressed a desire for simpler measures that are more compatible with current clinical practice [14]. Moreover, GPs and nurses expressed that the clinical evaluation of the elderly patient was complex. One aspect in particular was the inherent difficulty in distinguishing symptoms due to side effects of drug treatment versus those of chronic disease (box 1). The challenge of recognizing side effects of drug treatment has been described by GPs earlier [26]. In order to facilitate symptom evaluation in older patients, the guidelines on MRs (supplementary file) recommend the use of standardized questionnaires screening for common side effects of drug treatment [27]. However, GPs and nurses in our study experienced that the usefulness of such a questionnaire varied as they for example found it difficult to interpret the answers (box 4).

GPs and nurses disagreed with several aspects of the guidelines’ definition of MRs. The guidelines recommend MRs for all patients aged 75 and older with more than five drugs (supplementary file), whereas GPs and nurses in our study disagreed that age is a valuable reason to perform a MR (box 2). In a study by Sinnige et al. GPs expressed that they feel uncertain about which patients are eligible for a review [28]. Sinniges as well as our findings illustrate that it is important to define more thoroughly which patients are in need of a MR.

In contrast to the guidelines (supplementary file) GPs and nurses expressed that MRs are part of every consultation (box 2), and they questioned the differentiation between basic and complex MRs (box 2). This raises the question to what extent the guidelines’ definition of MRs and GPs’ and nurses’ definition actually overlap. In general, prescribers often describe negative attitudes towards guidelines, as they may feel the pressure to adopt them even if the guidelines do not fit clinical practice [12]. However, there are important arguments to stimulate the performance of regular MRs as a distinct measure apart from being “part of every consultation” (box 2), and thus to consider multimorbidity and polypharmacy as a distinct disease entity. First, multimorbidity is more common in elderly patients in primary care than single disease [29]. Second, GPs would be liberated from the uncertainty they experience in relation to their responsibility of the current drug list (box 2) [10] [30], as they would have a clear mandate to treat multimorbid patients instead of patients with a sum of several disease conditions. Third, GPs might dare to deprescribe more easily and accept that they ‘in a way preside over the subspecialist’ (box 2). Recommendations on the care of multimorbid elderly patients have recently been released by the National Institute for Health and Care Excellence as well as the The Royal college of GPs in UK [31,32]. Important suggestions are amongst others distinct MRs with a greater focus on the effectiveness of drug treatment; and that not a single disease itself but instead, the patient’s needs should be prioritized when optimizing drug treatment. In this context, an interesting attempt has been made by van Summeren et al. [33] who proposes the use of an “outcome prioritization tool for MRs”. Major effects of such a tool would be to involve patients in the decision-making process, but even to help prescribers to overcome their fear of being blamed for negative effects of deprescribing [32].

MRs as proposed by the guidelines are difficult to implement in primary care. GPs and nurses should participate in the construction and release of such guidelines in order to increase their usability in clinical practice. Furthermore, the multistep procedure should be condensed to the most important steps. Several former research findings may point towards how a MR may be facilitated in order to be applicable in clinical practice. First, during a MR it is necessary to focus on the detection of the most frequently encountered drug-related problems in elderly patients in primary care. A lack of indication and effectiveness of drug treatment as well as a too long duration of drug treatment accounted for more than one third of drug-related problems in elderly outpatients in Denmark [34] and the US [35]. In an Israeli study, MRs in elderly multimorbid patients consisted of checking the indication of treatment in relation to the patients’ age, comorbidity and possible adverse drug reactions [36]. As a consequence, in mean 4,4 drugs per patient could be removed, the participants’ quality of life increased, and dementia symptoms in three patients diminished. To assure both the indication and effectiveness of drug treatment must therefore be considered as the two most important steps during a MR minimizing a large proportion of all drug-related problems. Drug-drug interactions for instance are closely associated to the number of drugs which implies that they are reduced automatically when clinically unnecessary drugs are removed [37]. Second, the large majority of drug-related morbidity is caused by a limited number of PIMs such as anticoagulants, oral antiplatelet agents, non-steroidal anti-inflammatory drugs, hypoglycemic agents and central-nervous system acting drugs [38]. A Scottish study focused on feedback on prescribing of nine PIMs with the potential of serious side effects, namely gastrointestinal bleeding, renal failure and heart failure [39]. As a result, drug-related hospitalizations and PIM use decreased. This illustrates that it may be sufficient to screen for a limited but clinically relevant number of PIMs. Finally, it has been shown that certain drug-related problems are more common in presence of certain patterns of morbidity [40]. To give an example, patients with a combination of diabetes and renal impairment had a higher likelihood of drug-disease interactions than patients with diabetes only. The identification of more such morbidity patterns would enable clinicians to target MRs at patients at risk for drug-related problems.

Future research should analyse if condensed MRs and feedback on prescribing can be implemented in primary care, and if GPs and nurses experience that these are applicable and helpful measures in clinical practice. Furthermore, those affected by those measures should be enquired about their experiences. Finally, the effects of those measures on health-related patient outcomes should be evaluated.

## Conclusion

The pharmacists’ diaries showed that GPs and nurses should not be forced to perform MRs according to guidelines which do not fit clinical practice in primary care. Instead, GPs and nurses should take part in the construction and release of guidelines on MRs, in order to increase their acceptance amongst health care professionals as well as their usability in clinical work.

## Supplementary Material

Supplemental Material
