# Supplementary material for: General practitioners’ and nurses’ views on medication reviews and potentially inappropriate medicines in elderly patients – a qualitative study of reports by educating pharmacists
Source: Scand J Prim Health Care. 2018 Jun 29;36(3):329–41. doi: 10.1080/02813432.2018.1487458 (PMC6381541; doi:10.1080/02813432.2018.1487458)
Supplement: Supplemental Material [file IPRI_A_1487458_SM1614.docx]

*SUPPLEMENTARY FILE*

GUIDELINES ON MEDICATION REVIEWS ISSUED BY STOCKHOLM COUNTY

The guidelines were developed in relation to the update of the Swedish legislation year 2012 (1).

# Basic medication review

1. A basic medication review should be offered to
   1. all patients aged ≥75 at appointment with a GP or at hospital admission
   2. all patients independent of age at entry to nursing home or registration to home care (2)
2. Contents of a basic review
   1. update of current drug list with indication
   2. drug dose
   3. intake of drugs
   4. over-the-counter drugs/homeopathic drugs
   5. application of drugs, practical problems with intake, adherence/compliance
   6. in case the patient is not able to account for the drug list him/herself, a close person should be asked for information.
3. The GP/physician evaluates if the drug treatment is suitable and safe. If drug-related problems are suspected, they should be resolved immediately if possible. If there remain drug-related problems after a basic medication review, a comprehensive medication review should be offered.
4. After the basic medication review
   1. the GP/physician should document in the electronic patient record
      1. which drugs the patient uses
      2. which drug-related problems were discovered
      3. which measures have been undertaken and why
      4. how follow-up will be done
   2. the patient
      1. should be provided information on drug treatment and measures to be taken
      2. should be provided an updated current drug list.

# Complex medication review

1. A complex medication review should be offered
   1. annually to
      1. home care patients (2) and patients living in nursing homes
      2. patients aged ≥75 with multidose drug dispensing
      3. patients aged ≥75 with ≥ three chronic diseases
   2. other patients if drug-related problems are suspected or remain after a basic medication review
2. A complex medication review incorporates a basic medication review. It should be performed in team with patient, responsible physician, nurse, eventually pharmacist/clinical pharmacologist, other health personnel and contact person. The GP/physician performing the advanced medication review should evaluate the total current drug list.
3. Contents of a complex medication review
   1. update of current drug list
   2. screening for side effects with a standardized questionnaire (3)
   3. blood sample: hemoglobin, electrolytes, creatinine (…) (depending on patient´s diagnoses and drug treatment)
   4. blood pressure (optionally supine), pulse, weight
   5. estimation of renal function
   6. screening for drug-drug interactions with computerized decision support (4)
   7. falls in patient history?
4. For each drug, the following points should be checked:
   1. indication
   2. effect in relation to treatment target
   3. appropriateness in relation to patient´s age, diagnosis, renal function, other drugs
   4. drug dose in relation to patient´s age, diagnosis, renal function, other drugs
   5. evaluation of benefit and harm
   6. evaluation of non-pharmacologic alternatives
   7. drug formulation
5. For the total current drug list, the following points should be checked:
   1. drug treatment according to guidelines
   2. patient understands the drug treatment
   3. patients may handle drug treatment
   4. undertreatment
   5. clinically relevant interactions?
6. After a complex medication review
   1. GP/physician should document in the electronic patient record
      1. which drugs the patient uses
      2. targets of drug treatment
      3. which drug-related problems have been discovered, measures
      4. how and when follow-up should be performed, as well as who is responsible
   2. the patient should be provided
      1. information on
         1. which measures have been undertaken and why
         2. how and when follow-up should be done
         3. who is responsible for follow-up
         4. who has participated in medication review
      2. an updated current drug list.

**References**

1. The National Board of Health and Welfare (Socialstyrelsen). SOSFS 2012:9 Ändring i föreskrifterna och allmänna råden (SOSFS 2000:1) om läkemedelshantering i hälso- och sjukvården [Changes in regulations and general advices regarding the handling of drugs in the health care system]. Västerås; 2012.

2. Modin S, Furhoff AK. Care by general practitioners and district nurses of patients receiving home nursing: a study from suburban Stockholm. Scand J Prim Health Care. 2002;20(4):208-12.

3. Hedström M, Lidström B, Hulter Åsberg K. Phase-20: a new instrument for assessment of possible therapeutic drug-related symptoms among elderly in nursing homes. Vard Nord Utveckl Forsk. 2009;29(4):9-14.

4. Andersson ML, Bottiger Y, Lindh JD, Wettermark B, Eiermann B. Impact of the drug-drug interaction database SFINX on prevalence of potentially serious drug-drug interactions in primary health care. Eur J Clin Pharmacol. 2013;69(3):565-71.
